# Case of Congenital Opacity of the Cornea

**Published:** 1845-09

**Authors:** Philip W. Maclagan

**Affiliations:** Staff Assistant Surgeon


*Case of Congenital Opacity of the Cornea.* By PHILIP W. MACLAGAN, M. D., Staff Assistant Surgeon.—Mrs. K., wife of a private soldier in the Royal Canadian Rifle regiment, was delivered on the 7th of October, 1844, of a healthy female child. This was her fourth. The three others, all girls, bore evident marks of a dropsical constitution, but at the same time were healthy, good-looking children. As the woman belonged to a detachment some miles from my own residence, I was not with her during her labour; but next day, on my visit, my attention was called by one of the soldier's wives to the infant, which she said was *born blind.*

The state of the eyes at this time, *i. e.* about fourteen hours after its birth, was as follows:—On neither was there the slightest trace of vascularity or purulent discharge; the left cornea was completely opaque; the right was in the same condition, on its inferior two-thirds, but the upper third was clear, the opacity terminating by a tolerably defined edge. At first, I thought that I could perceive this edge to change its position, as the child's head was inclined to one side or the other, which led me to suppose the opacity resided in the aqueous humour; but this I found to be a mistake. Never having seen such a case, and not being able to hear of one, I was led to form an unfavourable prognosis: but in this I was agreeably disappointed; for in a few weeks the edge of the opacity on the right cornea began to thin off, to become less defined, and at length to recede, so that a part of the pupil could be seen on looking straight at the eye, while at first it could only be observed by looking from above. It was long before any change could be perceived on the left eye; but about the beginning of January, *i. e.* three months after birth, it too began to improve—the opacity at the upper part of the cornea becoming more diluted-looking, and by degrees disappearing. At this time, it was curious to observe the infant instinctively depressing the eyeball, when any bright object was held before it, so as to permit its image to fall through the upper portions of the cornea.

When I was removed from that post, a few days ago, the improvement was gradually progressing. There is now only a small portion of the right cornea opaque, and the upper half of the left is tolerably clear, so that the child directs the eyes forwards, and not as formerly, downwards; and I have great hopes that the opacity may disappear entirely, or at least so far as to leave vision totally unimpeded. She was vaccinated on February 12th, and soon after attacked by a very mild variolous epidemic, at that time prevalent in the neighbourhood, After this she had superficial ulceration behind the ears, which I rather encouraged than otherwise ; but it did not seem to have any influence on the clearing of the eyes. No treatment of any kind was employed.

Since I came to Kingston, I have had an opportunity of seeing ***Braithwaite's Retrospect***, in which I find several remarks on this curious subject ; but as the affection does not appear to be very common, I have thought that a relation of another case might be interesting. One thing, I think, may be drawn from all the cases recorded, viz., that the opacity has a great tendency to cure itself, if left alone.

*Lond. and Ed. Month. Journ. of Med. Sci.*

